# Individualized medical treatment options in Cushing disease

**DOI:** 10.3389/fendo.2022.1060884

**Published:** 2022-12-02

**Authors:** Aleksandra Gilis-Januszewska, Anna Bogusławska, Ewelina Rzepka, Witold Ziaja, Alicja Hubalewska-Dydejczyk

**Affiliations:** Chair and Department of Endocrinology, Jagiellonian University Medical College, Cracow, Poland

**Keywords:** Cushing disease, osilodrostat, pasireotide, temozolomide, ketoconazole

## Abstract

Cushing disease (CD) is caused by a pituitary tumor which oversecretes adrenocorticotropic hormone (ACTH). It is a serious endocrine disease associated with increased mortality and impaired quality of life. The management of CD remains challenging. Although transsphenoidal surgery is the treatment of choice in most cases, in approximately half of CD patients, second or third-line treatment options are needed. Currently, new medical therapies are available which target adrenal steroidogenesis, pituitary somatostatin and dopamine receptors, and glucocorticoid receptors. Selection of which medication to use should be individualized and is determined by many factors including severity of the disease, possible side effects, patients preferences and local availability. The aim of this article is to describe currently available medical therapy to help clinicians individualize the treatment options in the context of recently updated Pituitary Society recommendations.

## 1 Introduction

Cushing disease (CD), caused by overproduction of adrenocorticotropic hormone (ACTH) due to a pituitary tumor, was first described by Harvey Cushing in 1932 ⁠⁠ (1). This is a rare and serious endocrine disease associated with increased mortality and impaired quality of life. In the past, median survival time of CD patients was less than 5 years ⁠ (2). Despite newly available and effective methods of treatment, CD therapy and follow-up remains challenging. The main goals of treatment in CD are to normalize cortisol secretion, control the pituitary tumor, minimize clinical symptoms and comorbidities, improve the quality of life, and decrease mortality (3). Although transsphenoidal resection of a pituitary tumor remains the treatment of choice, approximately one-third of patients undergoing initial surgery may require second or third-line treatment options such as re-operation, radiotherapy, medical treatment, or bilateral adrenalectomy (4). Medical treatment is used in cases of persistent or recurrent hypercortisolism in patients who are not candidates for or refuse surgery, as bridge therapy during radiotherapy, as well as an initial therapy prior to surgery in severe hypercortisolemia (3). Currently, new medical therapies are available which target adrenal steroidogenesis, pituitary somatostatin, dopamine receptors, and glucocorticoid receptors. Additionally, there are novel experimental therapies under investigation such as chemotherapy and immunotherapy which might improve treatment outcomes. The selection of medical therapy should be individualized depending on the severity of disease, comorbidities, possible side effects, individual preferences, and local availability. The aim of this article is to summarize currently available medical treatment for Cushing disease with regard to the factors which should influence the choice of therapy in the context of recently updated Pituitary Society recommendations (3).

## 2 Epidemiology

Endogenous Cushing syndrome (CS) is a very rare disorder with an incidence of approximately 0.7–2.4 per million per year (5) ⁠⁠and a prevalence of 39-79 per million (6)⁠. Endogenous hypercortisolism is classified into ACTH-dependent (80-85%) and ACTH-independent (15-20%) CS⁠. Pituitary adenoma (also defined as pituitary neuroendocrine tumor (PitNET)) is the most common (75-80%) source of ACTH overproduction and this condition is defined traditionally as CD⁠. More than 90% of CD cases are caused by microadenomas (4, 7)⁠. In 40-50% of cases, clear visualization of the pituitary tumor may be challenging. Additionally, commonly occurring pituitary incidentalomas can hinder the diagnosis (4, 6)⁠. In a study based on the Swedish National Patient Registry (1987-2013), which included 502 identified patients with CD, the mean age at diagnosis was 43 years and women were more commonly affected than men (77% of cases) (8)⁠⁠. This data is similar to those found in recently published reviews⁠ (2, 5, 7)⁠. The standardized mortality ratio (SMR) was 2.5 in all patients. Moreover, it was still elevated even after achieving biochemical remission (SMR 1.9) (8).

## 3 Treatment

**Figure 1:**
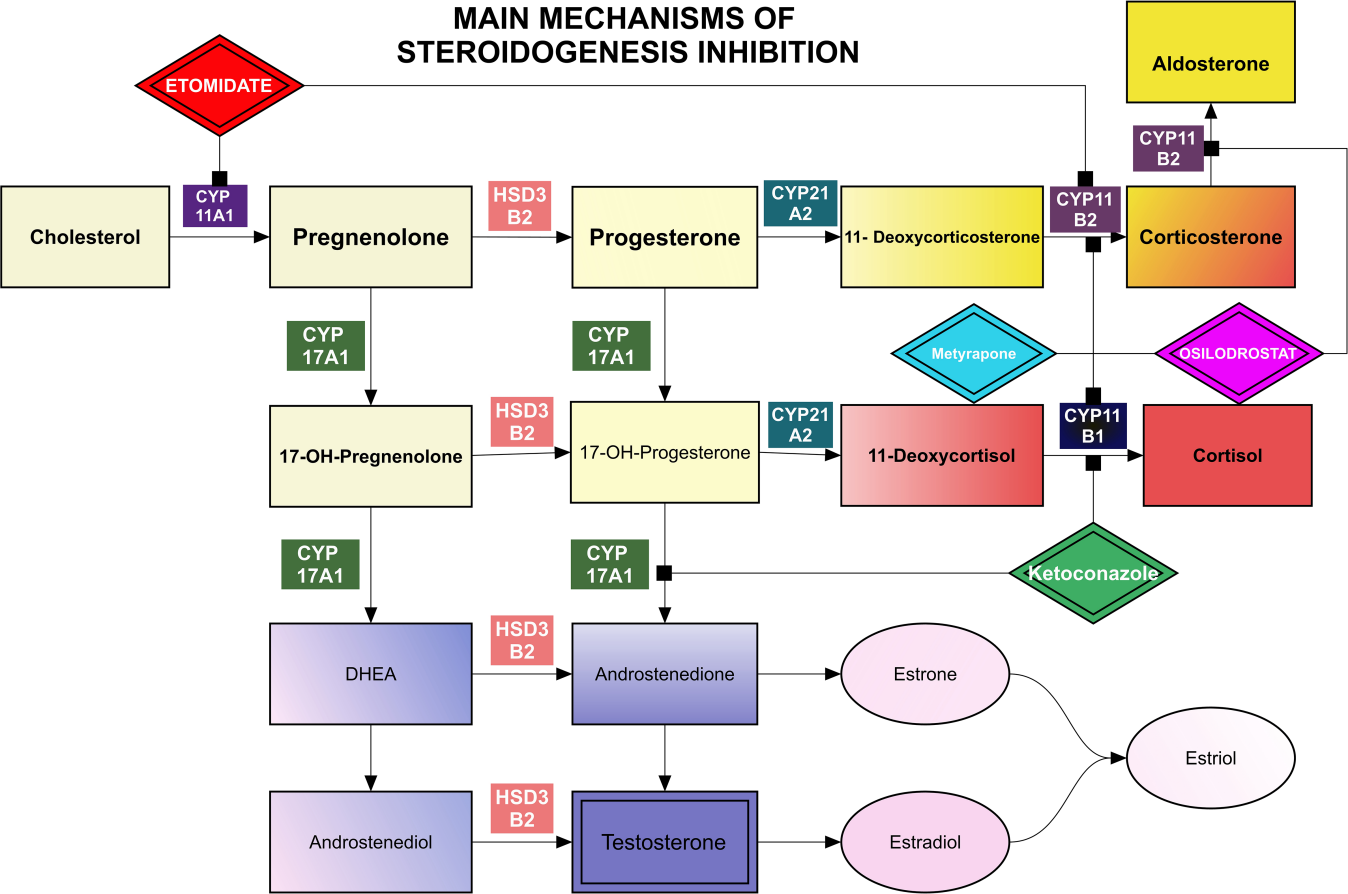
Main mechanisms of steroidogenesis inhibition.

After confirmation of a PitNET as the source of excessive ACTH production, surgery is the first-line treatment. Other possible therapies such as pharmacological treatment, radiotherapy, radiosurgery, and bilateral adrenalectomy have a supportive and complementary significance. Medical treatment may be used to control hypercortisolism as an initial treatment prior to surgery, bridge therapy following radiosurgery/radiotherapy, and also in situations when surgical resection of a corticotroph PitNET is contraindicated, not possible, not accepted, was non-curative, or when recurrence had occurred (2). Remission, defined as postoperative serum cortisol concentrations below 55 nmol/L (<2 μg/dL), is observed in 80% of patients with microadenomas and 60% of patients with macroadenomas (3). Transsphenoidal surgery (TSS) should be performed by an experienced surgeon with subsequent follow-up by a multidisciplinary team, including a pituitary endocrinologist. Current medications in use can be generally divided into 3 groups: inhibitors of steroidogenesis, tumor-directed drugs, and glucocorticoid receptor antagonists (**Figure 1**, **Tables 1**, **2**). In addition to the typical clinical signs related to hypercortisolism, PitNETs, especially those larger than 1 centimeter, may cause mass effect symptoms such as headaches, loss of vision, and pituitary insufficiency. Patients with ACTH-positive macroadenomas have a lower chance of being cured by transsphenoidal surgery (estimated to be approximately 12.5-71.1%), while microadenomas can be successfully removed in 65 - 95% of cases (32).⁠ Nevertheless, relapse occurs in 10-17% of patients (33)⁠, or even up to 30% of patients according to other authors (21).

**Table 1:** Current medications of Cushing disease with drug characteristic.

Medication	Efficacy(mUFC normalization)	Onset of action	Frequently used dose	Adverse events	Impact on sex hormones	% of population who discontinued/reduced treatment due to AE,intolerance, or other reasons)	Escapephenomenon(% of initiallyresponsive patients)
Ketoconazole	45-93% (4) M:64%	Few days	400-1600mg/day	-Hepatotoxicity 15% (4)⁠-Gastrointestinal disturbances 13% (4)⁠-Skin rash 5%-Adrenal insufficiency up to 20% (4)	Reappearance or improvement of menstrual cycles was reportedImprovement of hirsutism in some cases (4)	7–13%; M:11% (4)⁠	7–23%; M:15% (4)
							15% (9)⁠
Metyrapone	45.4–100%M:71%^4^	Fast	500-6000mg/day	Hirsutism: 36.1% hypokalemia:10.1%nausea, fatigue, dizziness, arthralgia (4)-Adrenal insufficiency 12% (10)	Hirsutism: 36.1% (4)	⁠0–13%; M:6% (4)	0–19%;M: 8% (4)
	64-94% (11)			Gastrointestinal complaints 23%, hypoandrogenism 7% (12)⁠⁠	Incidentally worsening of acne (12)⁠⁠	23% (12)⁠⁠	
Osilodrostat	66%-79%	Fast	4-14mg/day	nausea (42%), headache (34%), fatigue (29%) hirsutism (11%) acne (11%) hypertrichosis (1%) hypokalemia (13.1%) hypertension (12%), QT prolongation (4%), pituitary tumor enlargement (3%) potentially arrhythmogenic-related episodes (1%) (13) Hypocorticolism related AEs-27% (14)	hyperandrogenemia	18%-total11%- because of adverse events	Was not observed
Levoketoconazole	Early: 66-81%; after 6 months: 31-38% (15)	Few days	300-1200mg/day	-nausea (30 [32%])-headaches (26 [28%])- ALT >3x ULN (10[11%])-prolonged QT (2[2.1%])-adrenal insufficiency susp. (3[3.2%])	lack of data	18% (15)	**53%** (15)
Pasireotide	13-25% normalizationsubstantial reduction in 37.6%after 12 months: 28.4%	1-2 months	300-1800 ug/day	73% hyperglycemia20% gallstones8% adrenal insufficiency2% prolonged QT>480ms	Lack of data, probably beneficial influence on males and females	52%	24%
Pasireotide LAR	41-42% (16)	weeks/months	10-14mg/month	Hyperglycemia 47-49%Diarrhea 35-43%Cholelithiasis 15-45%diabetes mellitus 19-24%nausea 20-21%Serious AEs 5-11%	Lack of data, probably beneficial influence on males and females		
Cabergoline	36-40% (17, 18)⁠	weeks/months	0.5-7 mg/week	dizziness (14%), nausea (12%), asthenia (12%), dyspepsia (3%), abdominal pain, hypotension, muscle pain, alopecia and edema (2% each) (18)⁠	Probably beneficial influence on males and females	28% stopped (treatment escape or intolerance)	18–33% (11)⁠
Mifepristone	87% - clinical response	VERY FAST	300-1200mg daily	Hypokalemia – 44%endometrial thickening – 38%TSH elevation, thyroid hormones metabolism change – 19%QT prolongationinhibition of CYP3A4	Abortifacient	Lack of data	Lack of data
Etomidate		VERY FAST	0.02–0.1 mg/kg/h	adrenal insufficiency sedation, propylene glycol toxicity; plasma hyperosmolarity and lactic acidosis, central nervous system depression, cardiac arrythmias (19, 20)	Lack of data	Lack of data	Lack of data

**Table 2:** Individual approach depending on selected clinical problems.

Clinical problem	Suggested medication
Severe disease	• Osilodrostat-consider high initiating dose, fast dose increase, in selected cases block and replace regimen • Metyrapone • Etomidat- acute intravenous treatment/in case of i.v. action- (off-label use) • Combination therapy
Mild disease	• Pasireotide • Cabergoline • Osilodrostat- lower initial dose, slow up-titration, higher midnight dose in case of insomnia- can help to restore normal cortisol circadian rhytm (3)
Tumor shrinkage/residual tumor/large tumor remnant	• Pasireotide (21) • Cabergoline (22) • **Careful monitoring during treatment with**: osilodrostat (23), mifepriston (24), ketoconazole (9) • **In case of aggressive tumor**: temozolomide, immunotherapy (25)
Procreation plans/pregnancy	• Metyrapone (several studies) • Cabergoline (several studies) • **Not allowed:** mifepriston, mitotane • **I**n 1^st^ trimester avoid ketoconazol, levoketoconazol • In case of planning pregnancy discuss bilateral adrenalectomy
Hyperandrogenism in women	• Ketoconazole (26) • levoketoconazole • **Avoid:** metyrapone, osilodrostat (27, 28)
Hypogonadism in men	• Osilodrostat (28) • **Avoid:** ketoconazole
Liver impairment	• Osilodrostat (28, 29) • **Avoid:** ketoconazole (30)
QT prolongation	• **Careful monitoring during treatment with:** ketoconazole, levoketokonazol, metyrapon, osilodrosta, mifepriston, combination therapy with drugs potentially causing QT prolongation (e.g. ketokonazol and osilodrostat, osilodrostat and etomidat, pasireotide and osilodrostat, pasireotide and metyrapone) • Exclude potential interfering drugs • Electrocardiogram assessment before drug implementation is required
hypokaliemia	• **Careful monitoring during treatment with**: osilodrostat, metopiron (10, 31) • consider to add spironolactone
hyperglycemia	• **Careful monitoring during treatment with:** pasireotide (21)
Risk of CYP interaction	• **Careful monitoring during treatment with;** osilodrostat, relcorilant, ketoconazole, mifepristone

### 3.1 Inhibitors of steroidogenesis

#### 3.1.1 Ketoconazole

Ketoconazole is an imidazole derivative which was developed in 1977 and initially used as an anti-fungal drug (34). ⁠ It was considered to be effective, cheap, and well tolerated; however, it was later discovered to incidentally cause gynecomastia (35)⁠. This observation led to the conclusion that ketoconazole blocks cytochrome-P450 enzymes, causing a significant reduction in cortisol secretion. Subsequently, this led to its therapeutic use in the treatment of CD (35)⁠. ⁠ With important limitations such as hepatotoxicity, inhibition of androgen secretion in men, and gastrointestinal side effects, it was used as a valuable pre-surgical agent with a presumed efficacy of up to 50-70%; however, its effectiveness in long-term treatment was insufficient (9, 36)⁠⁠. Data from over 300 CS patients treated in five studies with a mean dose of 600 mg ketoconazole daily showed urinary free cortisol (mUFC) normalization in approximately 64% of cases after 12.6 months (4). However, over 20% of initially responsive patients lost biochemical control. A decrease in body weight and arterial hypertension along with improvement in glucose metabolism were noted. In 2013, the European Medicines Agency (EMA) and American Food and Drug Administration (FDA) questioned the clinical use of ketoconazole due to the risk of hepatotoxicity, potentially leading to liver transplantation or death (34)⁠. A meta-analysis of studies from 1979 to 2012 supported these observations, and estimated the risk of ketoconazole-associated hepatotoxicity to range from 3.6% (95% CI, 3.2-4.2) to 4.2% (95% CI, 3.7-4.9) (30)⁠. However, in a cohort study of 195 334 azole initiators published in 2015 by Lo Re et al. (37), the estimated risk of severe acute liver injury was not increased with the use of ketoconazole when compared with fluconazole. Hepatotoxicity in the course of ketoconazole treatment is asymptomatic in most cases and occurs mainly within the first 6 months of treatment. Elevations in liver enzymes do not appear to be dose-dependent and normalize by 12 weeks after discontinuation of the drug. Ketoconazole blocks multiple cytochromes, including those involved early in the steroid biosynthetic pathway such as the P450 enzymes, mainly the cholesterol side-chain cleavage complex, 17α-hydroxylase, 11β-hydroxylase, and aldosterone synthase. Ketoconazole was typically used in doses of 600-800 mg/24h (possible range of 200-1200 mg, up to 1600 mg⁠) (11), and due to its short half-life of approximately 3.3 hours, it must be administered at least 2-3 times per day (4). The remission rate was estimated to be approximately 44.7-92.9% (7)⁠. Typical side effects included hepatotoxicity (10.7-18.7%), skin rash (3.6-6.2%), adrenal insufficiency (5.3-18.2%), and QTc prolongation (7). In a study including 51 patients treated with ketoconazole for more than 24 months, the escape phenomenon was observed in 15.4% of initially responsive patients (9)⁠. Ketoconazole therapy for CS is off-label in the USA. Concomitant inhibition of androgen secretion suggests that it could be used as an alternative for women with CS and concomitant hyperandrogenemia.

#### 3.1.2 Levoketoconazole

Levoketoconazole is a substance purified from racemic ketoconazole and has been investigated as a potentially effective drug for the treatment of type 2 diabetes (13).⁠ It is more potent than the 2R, 4S enantiomer, especially in the inhibition of 11β-hydroxylase, 17α-hydroxylase, and CYP51A1, a critical enzyme for fungal survival (13, 38).**⁠^⁠^⁠** Preclinical studies in animals revealed that levoketoconazole is significantly more effective in corticosterone reduction than racemic ketoconazole (13)**⁠**. The SONICS study (phase 3, multi-center, open-label, non-randomized, single-arm study) investigated the safety and efficacy of levoketoconazole in CS patients (85% of cases with CD) (15, 39). In this study, patients received an oral form of the drug using an incremental dose-titration protocol starting at 150 mg twice daily until mUFC normalization or maximal dose (600 mg twice daily) was reached. Next, a six-month maintenance phase with a stable dose of medication was initiated, while mUFC at the beginning was approximately 5 times the upper limit of normal (ULN). Seventy-seven out of the 94 patients (82%) advanced to the maintenance phase. Among them, 81% achieved mUFC normalization, while after the six-month maintenance phase, 47% of patients presented with a normal mUFC value. Typical adverse events (AEs) included nausea (32%) and headaches (28%). Interestingly, 2 patients had a QT-interval >500 ms and 3 patients were suspected to have adrenal insufficiency. In 10 patients (11%), alanine transaminase (ALT) reversibly increased more than 3 times the ULN. The LOGICS study (double-blind, placebo-controlled, randomized-withdrawal study) was conducted after the SONICS study, and was completed in August 2020 (40). In total of 84 patient (12 after SONIC study) with the stable dose of levoketoconazole were included and individualized protocol of the drug titration was used. Patients with the therapeutic dose of levoketoconazol entered randomized withdraw (22 receiving the drug, 22 in placebo group). Loss of mUFC response in 95.5% of patients in placebo group compared to 40.9% of patients treated with levoketoconazol (P = 0.0002) was observed. AEs occurred in 89% of patients. Levoketoconazole appears to be a promising novel agent, but some serious AEs such as QT prolongation (10.7%) and increased ALT in a large percentage of patients must be taken into consideration (40). Additionally, a direct comparison with ketoconazole could provide more information on the usefulness of levoketoconazole. To date, no head-to-head studies in humans have been performed. Levoketoconazole has been recently approved by the FDA for the treatment of endogenous CS.

#### 3.1.3 Metyrapone

Metyrapone was first described as a cortisol-lowering agent in 1958 by Liddle et al. (38). It acts mainly *via* 11β-hydroxylase inhibition, but it also inhibits 18-hydroxylase (aldosterone synthase) (41). As a result of the reduction in cortisol-mediated negative feedback on corticotropic cells, ACTH oversecretion is observed, followed by the overproduction of androgens and mineralocorticoid precursors, especially 11-deoxycortisol. Metyrapone effectiveness, depending on dose and individual reaction is approximately 45.4–100% (4). Typical side-effects of metyrapone treatment are hirsutism (4.5-71.4%), acne, hypertension (48.4%), hypokalemia (6.7-13.6%), and edema (20%) (41). Hyperandrogenism-related symptoms can be substantial, limiting prolonged therapy in females. Fatigue (13.0-13.6%), dizziness (16.0-44.4%), arthralgia (8.7-18.2%), nausea (5.3-13.6%), and less frequently, abdominal pain and atopic dermatitis were also reported (41). Furthermore, elevated 11-deoxycortisol may also cross-react with cortisol immunoassays causing false increase of serum cortisol by approximately 20% (12). Usually, AEs appear shortly (<15 days) after initiation of therapy or dosage increase (12). Metyrapone has a half-life of 2 hours, thus it acts rapidly but shortly. Because of this, metyrapone must be administered typically 4 or 6 times per day. The typical dose of metyrapone is 750 – 2000 mg per day, but some authors suggest that the maximum dose may be up to 6 g/day (36). In week 12 of treatment in the PROMPT study (prospective study), 47% of patients (23/49) had normalized mUFC. The final median dose of metyrapone was 1500 (250 - 5500) mg/day. In about 66% of patients signs and symptoms of the disease improved or resolved. Median ACTH production increased by 11% from baseline. The most commonly observed AEs were nausea (24%), decreased appetite (18%), fatigue (14%), headache (10%), peripheral edema (6%), hypokalemia (6%), and hypertension (6%). QT prolongation was observed in 3 patients only, it was asymptomatic and didn’t exceed 480ms. Nevertheless, the potassium, blood pressure and ECG follow-up management is recommended regarding metyrapone therapy (10, 31). Transient adrenal insufficiency was observed in 6 (12%) patients. AE resulted in dose interruption or dose adjustment was observed in relatively small group of patients (14%, 7/50) (10). A limitation of prolonged metyrapone treatment is the requirement for multiple daily administrations and potential AEs. Because of this, it should be used as a short-term therapeutic option, especially in men (13⁠, 39). There are some published reports regarding metyrapone treatment in pregnant women and in children prior to the initiation of radiotherapy (42). Similar to ketoconazole, prolonged metyrapone therapy may lead to the escape phenomenon in up to 18.7% of patients (41).

Metyrapone is EMA approved for the treatment of endogenous CD, while in the USA it is used off-label.

#### 3.1.4 Osilodrostat

Initially, osilodrostat was considered as a potential therapy for hypertension, cardiac failure, and renal disease (43). In a proof-of-concept study from 2014, Bretagna et al. demonstrated the efficacy and tolerability of osilodrostat in 12 CD patients (19). It is a potent, reversible inhibitor of aldosterone synthase (CYP11B2), and to a lesser extent, 11β-hydroxylase (CYP11B1) (19, 31)⁠. Osilodrostat is orally bioavailable and could be administered twice a day, since it has a longer half-life than ketoconazole and metyrapone (nearly 4 hours). In a phase I study, which was focused on plasma and urinary aldosterone decrease, a simultaneous decline in baseline and ACTH-stimulated cortisol secretion was observed. Adverse effects such as weight loss (25%), postural tachycardia (25%), and mild hyponatremia (33%) were observed mainly due to decreased aldosterone production (13). The phase II studies, LINC1 and LINC2, demonstrated the safety and efficacy of osilodrostat in the treatment of CS. After 22 weeks of treatment, 78.9% of patients had achieved mUFC normalization. Seventy-five percent of women involved in the study had elevated testosterone levels, but only 28.6% of them reported hirsutism and/or acne during treatment. Mild hypokalemia occurred in 47.4% of patients. Other frequent AEs such as nausea, diarrhea, and asthenia seem to be associated with decreased cortisol secretion (13). In the phase III study, LINC3, which was dedicated to osilodrostat efficacy and safety assessment in a large group of patients, normalization of mUFC was observed in 66.4% of cases at the end of the trial. Moreover, the metabolic-related parameters (including body weight, glucose metabolism, lipid profile), as well as blood pressure, quality of life, and depression status improved mainly during the first 12 weeks of treatment (13, 23). The proportions of patients with pituitary tumour growth in comparison to patients with tumor size decrease on osilodrostat were similar (33 vs 38%, respectively) (23).Results from a phase III study (LINC 4) with an initial placebo-controlled phase confirmed the efficacy of osilodrostat in normalizing mUFC at week 12, in comparison to placebo (77% vs 8%). The decrease in mUFC levels persisted throughout 36 weeks of treatment. The most common AEs were arthralgia (45%), decreased appetite (45%), fatigue (38%), nausea (37%), and headache (33%). AEs connected with adrenal insufficiency were infrequent and occurred in 15% of patients, while AEs associated with hormone-precursor accumulation were seen in 44% of patients. The study confirmed significant improvement in cardiovascular and metabolic-related clinical parameters, such as systolic and diastolic blood pressure and HbA1c concentration during treatment (14). In comparison to previous pharmacological treatment options in CS, osilodrostat has some unique advantages, such as less serious AEs when compared to ketoconazole and a longer half-life than ketoconazole and metyrapone. In recently published study by Detomas et al. comparing osilodrostat and metyrapone, both drugs showed similar therapeutic efficacy, however osilodrostat seems to reduce cortisol levels and to control blood pressure faster (44). Some additional differences in the steroidogenic enzymes inhibition between osilodrostat and metyrapone were also observed by Bonnet-Serrano et al. The authors showed smaller increase in 11-deoxycortisol and androgen levels in patients treated with osilodrostat in comparison with metyrapone therapy (45). It is important to note that a recent publication by Castinetti et al. (20) reported delayed, reversible adrenal insufficiency in 3 patients using a stable dose of osilodrostat highlighting the need for detailed patient education regarding potential side effects and careful follow-up during osilodrostat treatment (20). Similarly to metyrapone, in case of AI suspicion, clinical signs and symptoms should be carefully assessed due to risk of cortisol overestimation as a consequence of lab test cross-reactivity with some adrenal precursors proximal to the 11β-hydroxylase enzyme blockade (3, 31). Unless severe CS, slow up-titration of osilodrostat is recommended, which may decrease the risk of adrenal insufficiency (31). Since restoring normal circadian rhythm of cortisol is one of the aims of the CS treatment, a higher dose of osilodrostat at nighttime in patients with insomnia seems to be beneficial (3, 31). In patients with severe CS, rapid cortisol normalization can be achieved by block and replace approach, which facilitate fast dose increase, minimizing at the same time the risk of AI (20, 31).

Moreover, all patients before starting the treatment with osilodrostat should undergo potassium, magnesium assessment and ECG measurement. Electrolyte disturbances are needed to be corrected before initiation of the therapy. Further monitoring of those parameters to avoid hypokalemia and prolonged QT is also very important. For ECG, repetitive assessment at baseline, one week and 2-4 weeks after starting osilodrostat is proposed. Follow-up continuation should be determined by the clinical situation. In case of hypokalemia, apart from potassium adequate supplementation, additional implementation of mineralocorticoid receptor antagonists should be considered (31). Hypokalemia and QT prolongation during osilodrostat therapy are rare, they were reported in 13% and 4% of cases, respectively. However, some cases with grade 3 and 4 hypokalemia were noted and required dose interruption (23).

Since osilodrostat is a weak inhibitor of cytochrome P450 3A4 (CYP3A4), careful review of patient’s concomitant medications to avoid potential drug-drug interactions is important before starting the treatment and during follow-up (31).

Osilodrostat is now approved by the FDA and EMA for the treatment of CS.

#### 3.1.5 Etomidate

Etomidate, an imidazole derivative was developed in 1964. It was introduced into use in 1972 as a short-acting, intravenous non-barbiturate, hypnotic anesthetic induction agent (46). Its suppression of adrenocortical function was revealed during investigation of a significant increase in mortality in mechanically ventilated trauma patients who received etomidate infusions for sedation (47). One of the earliest studies demonstrating the clinical effect of low-dose etomidate infusion on steroidogenesis inhibition was reported in 1990 (27). It showed a dose-dependent reduction in cortisol concentrations during etomidate administration and reversibility of the effect following drug withdrawal. The main model of action of etomidate on steroidogenesis is the mitochondrial cytochrome p450-dependent adrenal enzyme 11β-hydroxylase inhibition; however, at higher concentrations it also inhibits the cholesterol side-chain cleavage enzyme. It may also affect aldosterone synthase and have an anti-proliferative effect on adrenal cortical cells (48, 49). The plasma half-life of etomidate is 3-5 hours (50). Etomidate is the only steroidogenesis inhibitor which may be administered parenterally. Currently, it is being proposed as an effective and safe therapy for severe hypercortisolemia when rapid control of cortisol levels is required, especially in patients who are intolerant to or unable to take oral medication (48, 51). The proposed intravenous dose of etomidate in intensive care unit (ICU) patients is 0.04–0.1 mg/kg/h. A low-dose etomidate infusion (0.02-0.04mg/kg/h, mean dose 0.025mg/kg/h), for the treatment of hypercortisolemia can be also effective in achieving target cortisol levels without the need for intensive care resources and with no risk of inducing adrenal insufficiency (31, 50, 52). A dose should be titrated according to serum cortisol concentration. A decrease in cortisol level is fast and occurs within 12-24h after drug implementation. Frequent monitoring is necessary to avoid adrenal insufficiency. The proposed target serum cortisol based on mean 24-h cortisol levels in intensive care unit (ICU) patients is 500-800 nmol/l, while the accepted range for serum cortisol in a non-acute setting is 150-300 nmol/l. However, reliable etomidate dose titration should rely also on patient’s clinical status careful assessment (50). The observed side effects during etomidate treatment include adrenal insufficiency (which could persist several weeks after drug discontinuation due to accumulation of the agent in subcutaneous tissue) (48, 53), sedation (usually observed at higher doses than those used to suppress cortisol production), propylene glycol toxicity (which is added because of etomidate hydrophobicity), including plasma hyperosmolarity and lactic acidosis, central nervous system depression, cardiac arrythmias. Side effects are observed mostly with prolonged etomidate infusion. The drug is degraded by hepatic enzymes but very rarely causes significant hepatotoxicity. In patients with renal failure, in severe condition, or in the elderly, the dose of etomidate should be decreased because of impaired protein binding and renal clearance (54). The main advantages of etomidate in CS therapy are its efficacy, rapid onset of action, and parenteral route of administration. Despite being in clinical use, etomidate is not FDA approved for the treatment of CS.

### 3.2 Pituitary-directed medications

#### 3.2.1 Pasireotide

Corticotroph PitNETs express somatostatin receptors (SSTR), mainly subtype 5 (SSTR5), followed by subtype 2 (SSTR2) and subtype 1 (SSTR1), providing potential therapeutic targets (21, 55). The first trials with octreotide were unsuccessful; however, the development of pasireotide provided clinicians with another potential treatment for PitNETs. Chronic exposure to high cortisol concentrations leads to down-regulation of SSTR2, limiting the effectiveness of octreotide therapy (13)⁠. Pasireotide is a novel somatostatin analogue administered subcutaneously. It has greater affinity for SSTR1 (30x), SSTR3 (5x), and SSTR5 (40x) than the classic somatostatin analogue, octreotide (56). In a 12-month phase III, double-blind study, 162 CD cases with mUFC ≥1.5-fold the ULN were randomly divided into 2 cohorts. Eighty-two patients received 600 µg of pasireotide twice daily, while 80 patients received 900 µg twice daily (21). After 3 months, some patients with poor reaction to pasireotide (mUFC >2xULN) had a dosage increase of 300 ug twice daily. At month 6, patients entered an open-label phase that lasted through month 12, and in this phase, the dosage could be increased up to 900/1200 µg twice daily. Forty-eight percent of patients completed the study and the median duration time was 10.8 months. After 1 year, 13% of patients in the 600 µg group and 25% of patients in the 900 µg group had mUFC levels at or below the ULN. Partial control of hypercortisolism was achieved in 18% and 13% of patients in the 600 µg and 900 µg groups at month 6, and in 16% and 3% of patients at month 12, respectively. Reduction in mUFC was rather rapid and sustained, suggesting a possibility to predict patient reaction to treatment at its early stage. Significant reductions in systolic blood pressure (−6.1 mm Hg), diastolic blood pressure (−3.7 mm Hg), triglycerides (–2 mg per deciliter), LDL cholesterol (-0.4 mmol), and weight (-6.7 kg) were observed. The health-related quality of life score also improved. Importantly, PitNET shrinkage was noticed: –9.1% of initial volume in the 600 ug group (95% CI, –46.3 to 28.0) and –43.8% (95% CI, –68.4 to –19.2) in the 900 µg group. The main AE observed was hyperglycemia, despite its safety profile being similar to other somatostatin analogues. Seventy-three percent of patients had a hyperglycemic-related AE and treatment was discontinued in 6% of all patients because of this reason. Moreover, an antidiabetic drug was administered to 46% of patients, while 64% of patients who were previously treated for diabetes had to receive another antidiabetic medication. Eight percent of patients had symptoms of adrenal insufficiency and 2% had a prolonged QT-interval of more than 480 ms. Elevation of ALT and aspartate aminotransferase (AST) more than 3xULN was not observed. Approximately 20% of patients with a normal gallbladder at baseline developed gallstones, while 4% of patients required a cholecystectomy. In summary, the study demonstrated the effectiveness of pasireotide. Approximately 50% of patients had a substantial reduction in mUFC, while reduction in tumor size was also noted. However, many patients developed hyperglycemia, which required additional treatment. This is the main disadvantage of pasireotide. Some observational studies suggest a higher efficacy of pasireotide in real-life settings, even up to 80%, especially in mildly to moderately severe patients. Other investigators have noted similar results to the phase III clinical trial (13).

#### 3.2.2 Pasireotide LAR

Pasireotide LAR is a modified, long-acting version of pasireotide, which is administered once monthly. It has similar efficacy and safety as the short-acting form, which was confirmed in a multicenter, randomized, double-blind, clinical trial (clinicaltrials.gov code: NCT01374906) (16). In this study, 150 patients with CD were treated with a 10 mg or 30 mg monthly intramuscular injection of long-acting pasireotide. In the 7^th^ month, 40% percent of patients showed normalization of mUFC, regardless of the dose and dose titration. Notably, the response was much higher in patients with less active disease (mUFC less than 2 times ULN; 52% in both the 10 and 30 mg group of pasireotide LAR) when compared to 35% and 37% in patients with mUFC of 2-5 times ULN (16). The median tumor volume decreased by 17.8% and 16.3% with doses of 10 and 30 mg respectively. A 20% reduction in median tumor volume was observed in 43% and 47% of patients receiving 10 and 30 mg doses respectively. Pasireotide is the only pituitary-directed medication approved by the EMA to treat CD in patients for whom pituitary tumor removal is not an option or for whom neurosurgery has failed or has not been curative (13).

#### 3.2.3 Cabergoline

Cabergoline is a potent dopamine 2 receptor (D2R) agonist, an ergot derivate, and is widely used in the treatment of hyperprolactinemia. Several studies have investigated its efficacy and safety in the treatment of CD patients. In a recent meta-analysis by Broersen et al., cabergoline efficacy was estimated to be approximately 36% (17). Interestingly, some publications suggest that some corticotroph PitNETs may be highly susceptible to dopamine agonist treatment (13)**⁠.** A recent retrospective, multi-center study from France and Belgium showed mUFC normalization in 40% of patients who received cabergoline as monotherapy (18)⁠. The fall in mUFC was associated with clinical improvement along with significant reductions in midnight cortisol and plasma ACTH levels. Nevertheless, after long-term treatment (>12 months), 28% of responders discontinued therapy due to treatment escape or intolerance. Finally, 20-25% of patients were specified as “good responders”. In addition, the study did not find any predictors of response to cabergoline. The dose of cabergoline used in the treatment of endogenous hypercortisolemia varies between 0.5-7 mg weekly (18, 57).

#### 3.2.4 Temozolomide

Temozolomide is an imidazotetrazine derivate and alkylating agent used in the treatment of malignant gliomas (anaplastic astrocytoma and malignant glioma), which can be administered orally or intravenously. It causes DNA methylation and alkylation, and its efficacy is limited by methylguanine DNA methyl transferase expression, a DNA-repairing enzyme which counteracts temozolomide’s antineoplastic action. Temozolomide causes cell injury, and as a result, leads to apoptosis and tumor shrinkage. Based on European cohorts (25, 58–60), temozolomide has been used as rescue therapy in aggressive or malignant PitNETs with promising results and good tolerance. The first case reports were described in 2006 (61), and to date, more than 150 patients with aggressive pituitary tumors have been treated (62, 63). Response (complete remission, partial response, or stable disease) was achieved in 69% of patients (33-86%). Tumor shrinkage or control of its size was observed in 55-70% of patients (11). Temozolomide may be a valuable option in a small subgroup of aggressive corticotroph PitNETs, especially Crooke cell tumors (63). Some data suggest the possibility of achieving long-term remission even after discontinuation of treatment (64)⁠. Some AEs of temozolomide therapy include anorexia, headaches, syncope, somnolence, and gastrointestinal symptoms such as nausea, vomiting (65–67). Recently, temozolomide monotherapy in standard doses was recommended by the European Society of Endocrinology as a first-line treatment for recurrent, aggressive PitNETs or pituitary carcinomas (62). In a recent paper in a large cohort of aggressive pituitary tumors/pituitary carcinomas, temozolomide was used in 156 out of 171 patients. The treatment with temozolomide resulted in complete response in 9.5% of cases. Partial response was observed in 30.1% cases, stable disease in 28.1%, and progressive disease in 32.2% of patients (25). However, still there are no randomized prospective trials or case-control studies. Evaluation of methylguanine DNA methyl transferase expression status may predict treatment response (62).

### 3.3 Glucocorticoid receptor antagonists and new possible therapies

#### 3.3.1 Mifepristone

Mifepristone is a potent progesterone antagonist and was developed as a medication for hypercortisolism in the early 1980s. It is a competitive glucocorticoid receptor antagonist, causing amelioration of CS symptoms. However, a lack of measurable markers of its action restricts its usefulness (6)⁠. Due to its antiprogesterone action, it may cause excessive bleeding, abortion, and endometrial thickening. Moreover, it can cause adrenal insufficiency with typical symptoms such as nausea, vomiting, headaches, fatigue, and arthralgias due to increased mineralocorticoid effects (hypertension, hypokalemia, edema) (6).⁠ Thyroid function changes have also been reported, such as unmasking of autoimmune thyroiditis (68). Moreover, alteration of thyroid hormone metabolism leading to the requirement of increased levothyroxine dosage in replacement therapy was observed, but the mechanism of this effect is still unclear (69). Mifepristone is metabolized by CYP3A4 and inhibits CYP2C8/9, which potentially may lead to interactions with a great number of medications. Concomitant therapy with simvastatin, lovastatin, and fentanyl is not recommended, while other medications may require a dose reduction. Mifepristone prolongs the QT-interval in a dose-dependent manner (68). These features imply the necessity of close monitoring and thoughtful dose adjustment in patients treated with mifepristone. Some data have demonstrated the efficacy of mifepristone in the treatment of endogenous hypercortisolism. The Study of the Efficacy and Safety of Mifepristone in the Treatment of Endogenous Cushing Syndrome (SEISMIC) investigated the efficacy of mifepristone and found that a clinically significant improvement was achieved in 87% of patients. The most common AEs were hypokalemia (44%), endometrial thickening (38% of women), and thyroid dysfunction (reversible elevation of TSH - 19%) (68). In SEISMIC, 28% (14/50) of patients received spironolactone at doses of up to 400 mg daily (68). In a study by Wannachalee et al., mifepristone efficacy in the treatment of ectopic ACTH-dependent hypercortisolism was comparable to bilateral adrenalectomy (70). In summary, mifepristone therapy, along with anti-mineralocorticoid medication is an option in some cases, especially in patients who cannot undergo operation or in cases of severe hypercortisolism where rapid treatment is necessary. In 2012, the FDA approved mifepristone for the treatment of hyperglycemia secondary to hypercortisolism in CS patients with type 2 diabetes mellitus or glucose intolerance and who have had unsuccessful surgery or are not candidates for surgery (68).

#### 3.3.2 Relacorilant

Relacorilant (CORT125134) is administered orally and is a highly-selective, non-steroidal modulator of the glucocorticoid receptor (71). It competitively blocks the glucocorticoid receptor by preventing translocation of the ligand-GR complexes to the nucleus and GR-associated gene transcription (72).⁠ Relacorilant is now under investigation in a phase III study (NCT03697109) (73). Importantly, it has no effect on the progesterone receptor (in contrast to mifepristone) and consequently does not cause termination of pregnancy, excessive menstrual bleeding, or endometrial hypertrophy (74). Phase I studies demonstrated the safety of relacorilant and its ability to ameliorate the pharmacological effects of prednisone (74).⁠ Similar to mifepristone, relacorilant strongly inhibits CYP3A4. In a pharmacological study, the presence of relacorilant led to a greater than 8-fold increase in midazolam overall exposure (51)⁠. Nevertheless, relacorilant does not inhibit CYP2C8/9 *in vivo* (75). Due to its long half-life (11-19 h) relacorilant may be administered once daily. It is rapidly absorbed; however, food intake may cause a delay in its absorption. Relacorilant appears to be safe; however, some mild AEs (predominantly musculoskeletal disorders) such as back pain were observed. Furthermore, it does not prolong QT-interval (74). In a recent phase II study, patients were divided into 2 groups - low and high dose receivers (76). Improvement in hypertension control was observed in 41.7% (low dose) -63.6% (high dose), while glucose metabolism was better in 15.4% of low dose and 50% of high dose receivers. Finally, a decrease in body weight was reported in 35.3% of patients receiving low dose and 60% of patients in the high dose group. Notably, mild AEs such as back pain (31.4%), headaches (25.7%), and peripheral edema (25.7%) along with a lack of drug-induced vaginal bleeding or hypokalemia suggest that relacorilant is relatively safe. Interestingly, some clinical observations showed that in patients with ACTH-secreting NETs, relacorilant may improve sensitivity of tumors to endogenous somatostatin and/or somatostatin analogs. In 2 patients with ectopic ACTH-secreting bronchial tumors treated with relacorilant in two clinical studies (NCT02804750 and NCT02762981), increased radiotracer uptake was observed after initiation of relacorilant in SSTR2-based imaging, without an increase in tumor size, when compared to pre-treatment results. Moreover, in 2 patients with CD who were treated with relacorilant before planned pituitary surgery, magnetic resonance imaging performed after a 3-month course of relacorilant therapy showed a regression of tumor size (tumor volume decreased from 155 mm^3^ to 84 mm^3^ in one case and from 7.2 mm^3^ to 4.4 mm^3^ in another). The possible effects of endogenous somatostatin on tumor tissue with up-regulated SSTR2 has been previously discussed (71). Taken together, relacorilant appears to be a promising glucocorticoid receptor antagonist with comparable efficacy to mifepristone but without its disadvantages. Additionally, its potential effect on pituitary tumor size and SSTR2 expression in tumor tissue may be an essential and interesting therapeutic option for patients having ACTH-producing NETs with SSTR2 desensitization and as pre-surgical treatment in cases of large PitNETs which cannot be radically operated. Ongoing preclinical studies in human pituitary cell lines and the phase III study of relacorilant in CS patients, which includes tumor imaging, may provide additional insight.

#### 3.3.3 R-roscovitine

R-roscovitine is a member of the cyclin-dependent kinase inhibitors. Since 1997 it has been tested on more than 100 neoplastic cell lines, demonstrating its efficacy in lung cancer, breast cancer, neuroblastoma, glioma, colon cancer, and other malignant neoplasms (77). Recently, it has been suggested that roscovitine may be an effective medication in corticotroph PitNET treatment due to inhibition of CDK/cyclin complexes. Tests using animal models have been promising and showed proopiomelanocortin (POMC) gene suppression leading to a reduction in ACTH secretion and cell proliferation. Additionally, human corticotroph PitNET cultures were susceptible to treatment with roscovitine. In 2018, a phase II study was completed and results were recently published. Nine patients with CD were treated with an oral form of roscovitine, 400 mg twice daily for 4 days each week for 28 days. None of the patients achieved primary endpoint (normalization of mUFC; ≤ 50 µg/24 h), however in three and two of them, ≥50% and 48% reduction of mUFC was noted respectively. In one third of patients AEs related to elevated liver enzymes, anemia and elevated creatinine level were observed (78).

#### 3.3.4 Immunotherapy

Hypophysitis caused by administration of ipilimumab (CTLA-4 antibody) suggested the possibility of using immunotherapy in the treatment of PitNETs, especially in aggressive subtypes. It was observed that the tumor immune microenvironment (TIM) plays a substantial role in tumor development, growth, and invasiveness. Pituitary neuroendocrine tumors may be influenced by the TIM through alteration of its features and could also provide a potential therapeutic window (79)⁠. Lately, the potential use of immune checkpoint inhibitors (ICIs) has provided some hope for patients who were ineffectively treated with temozolomide (62). However, only a few cases of corticotroph PitNETs treated with ICIs have been described in the literature thus far (62, 80–83). The first case was that of a 35-year-old woman with an aggressive ACTH-secreting PitNET. She initially responded to combined therapy with temozolomide and capecitabine, but further observation revealed liver metastases. Consecutive therapy with ipilimumab (3 mg/kg every 3 weeks) and nivolumab (1 mg/kg every 3 weeks) caused a reduction in tumor volume of the dominant liver metastasis by 92% and regression of the recurrent intracranial disease by 59%. At the same time, her plasma ACTH level decreased. The next 2 cases with functioning corticotroph pituitary carcinomas were treated as part of a phase II clinical trial with pembrolizumab (80). Two patients with functioning corticotroph pituitary carcinomas (not responding to surgery, radiotherapy, and chemotherapy), treated with pembrolizumab (200mg every 3 weeks), had partial radiographic (60% and 32%, respectively, according to Immune-Related Response Evaluation Criteria in Solid Tumors), and hormonal responses (80). The third case described a patient with a corticotroph carcinoma treated with ipilimumab (1 mg/kg) and nivolumab (3 mg/kg) every three weeks, followed by maintenance treatment with nivolumab (3 mg/kg every 2 weeks). This patient had a partial biochemical response and dissociated radiological response with decreases in the pituitary mass and pre-existing liver metastases. Nevertheless, there was a new liver metastatic focus. The maintenance phase with nivolumab stabilized disease regarding the initial liver metastases, however, a progression of newly visualized metastasis (effectively treated with radiofrequency ablation) and pituitary mass was observed. Caccese et al. (82) presented the case of a 47-year-old man diagnosed with an ACTH-silent non-functioning pituitary adenoma. The patient was treated with pembrolizumab (200 mg every 3 weeks) due to tumor progression many years after stereotactic radiotherapy and after an initial transient response to pasireotide and temozolomide. After 4 cycles of therapy, pembrolizumab was discontinued due to biochemical and radiological progression of the disease (82).

A clinical and pathological characteristics and treatment outcomes in a large cohort including 171 patients with aggressive pituitary tumors and pituitary carcinomas were recently published (25). Adrenocorticotrophic hormone (ACTH)-secreting tumors constituted 30% of all presented cases. Immune checkpoint inhibitors were used as second-line treatment in six patients, five of whom were ACTH-secreting PitNETs [two patients were presented previously by Cassese et al. (82) and Duhamel et al. (83)]. The outcomes of ICIs treatment in the presented group were not satisfactory– in all cases disease progression were reported (25). Nevertheless, in general, clinically significant responses (at least SD of longer duration) of ICIs treatment in aggressive PitNETs were observed in 7 out of 15 cases published in the literature (25).

Immune checkpoint inhibitors may be a promising therapeutic option for patients with aggressive CD who do not respond to standard pharmacotherapy with temozolomide; however, further clinical trials are needed to determine the subgroups of patients who will respond to such treatment. Two ongoing clinical trials (NCT02834013, NCT04042753) may provide new insight on the role of ICI in the treatment algorithm.

### 3.4 Combination therapy

Combination therapies thanks to additive or synergistic effects on ACTH and cortisol secretion of involving medications, could result in better control of hypercortisolism, lower risk of side-effects due to lower doses of therapeutic agents and possibly a lower rate of treatment escapes. However, there have been only a few studies and case reports regarding specific combination therapy (84–87). In a study investigating the combined treatment of ketoconazole (400–1 200 mg/day), metyrapone (3–4.5 g/day), and mitotane (3–5 g/day) involving 11 severe CS patients, including 4 with CD, mUFC normalization (mUFC ≤ ULN) was observed in 7 (63.6%) patients after 24–48 h. UC remained low to normal during the whole period of combination therapy. Clinical improvement was also noted in these patients. The most frequently reported AEs were hypokalemia (100%), increase in liver enzymes (81.8%), nausea and vomiting (63.6%) (84). In a study by Feelders et al., combination therapy with pasireotide (300–750 μg/day), cabergoline (1.5–4.5 mg/week), and ketoconazole (600 mg/day) was used in 17 CD patients. Here, mUFC normalization (mUFC ≤ ULN) was observed in 15 (88.2%) patients accompanied by clinical improvement. A gradual escalation of treatment regimen was performed, starting with pasireotide as the initial treatment and followed by cabergoline and ketoconazole if the previous first-line or second-line treatment at maximum doses failed to normalize mUFC (85). The CAPACITY study (phase II clinical study clinicaltrials.gov code: NCT01915303) assessed the efficacy and safety of combination therapy with pasireotide and cabergoline,. Pasireotide (1 200–1 800 μg/day) was administered subcutaneously twice-daily and cabergoline was administered once-daily (0.5–1 mg/day) in a group of 68 CD patients with mUFC > ULN, with or without preceding surgery. The group of patients consisted of pasireotide-naïve cases and those who previously discontinued the treatment for reasons other than AEs. Fifty percent of patients (52) achieved mUC normalization with pasireotide monotherapy, while 25% (41) required combination therapy. Clinical parameters as well as quality of life significantly improved. The most commonly observed AEs were hyperglycemia (51.5%), nausea (51.5%), and diarrhea (44.1%). Therapy withdrawal was reported in 16 (23.5%) patients. It was connected with AEs in 8 (11.8%) patients and a lack of efficacy in 3 (4.4%) patients. The final results of the study have not yet been published (86). In recently reported study a successful and well tolerated treatment with a combination of osilodrostat and etomidate in a severe case of CD in young female has been described (88).

Combination therapy with ketoconazole and osilodrostat, appearing to act in a synergistic manner was also described recently as efficient and well-tolerated strategy to suppress cortisol levels (89). The authors suggest that this strategy could be considered for any patient with uncontrolled hypercortisolism and delayed or unsuccessful surgery, also in terms of decreasing the cost of the therapy.

Further studies regarding combination therapy are needed. Finally, the possible risk of potential AEs, such as QTc prolongation, should be considered when selecting the treatment method.

## 4 Conclusions

In the treatment of CD patients, normalization of cortisol secretion is essential to prevent greater morbidity and mortality. Transsphenoidal resection of PitNETs remains the treatment of choice in CD. Medical treatment may be used as preparation for surgery or as bridge therapy after radiosurgery/radiotherapy, and in cases where surgical resection of a corticotroph PitNET is not feasible or recurrence has occurred. Currently, there are several medications available. Steroidogenesis inhibitors are effective in the management of hypercortisolism but may lead to some specific AEs. Pituitary-directed medications may lead to corticotroph PitNET shrinkage, but they are less effective in a severe disease. Cabergoline appears to be exceptionally efficient in a small group of patients, but there are no known markers to predict the response to treatment. Glucocorticoid receptor antagonists appear to be safe and effective; however, a lack of measurable markers of their action limits their usefulness. Temozolomide is the treatment of choice in aggressive CD. Immunotherapy is a novel approach in the treatment of aggressive PitNETs. Further studies are required to determine its potential in the treatment of aggressive CD. Therapy in CD patients following non-curative surgery should be individualized. The possible options and their combinations may lead to different strategies of treatment, providing additional benefits to patients.⁠

## Author contributions

AG-J and AB contributed equally to this work. AG-J contributed to the conception of the work. AG-J, AB, ER, WZ, and AH-D searched and extracted data. AG-J, AB, ER, and WZ drafted the manuscript and contributed to the acquisition, analyses, and interpretation. AG-J and AH-D critically revised the manuscript and contributed to interpretation. All authors have given their final approval and agree to be accountable for all aspects of the work, ensuring integrity and accuracy.

## Conflict of interest

The authors declare that the research was conducted in the absence of any commercial or financial relationships that could be construed as a potential conflict of interest.

## Publisher’s note

All claims expressed in this article are solely those of the authors and do not necessarily represent those of their affiliated organizations, or those of the publisher, the editors and the reviewers. Any product that may be evaluated in this article, or claim that may be made by its manufacturer, is not guaranteed or endorsed by the publisher.
